# Cultural Guideposts of Health: A crisis response evaluation framework for California’s diverse Indigenous communities

**DOI:** 10.1177/22799036251410263

**Published:** 2026-01-20

**Authors:** Krista Armenta-Belen, LittleDove Rey, Joshua Severns, Daniel Dickerson, Virginia Hedrick, Gloria Miele, Mamta Bhakta, Beth Rutkowski, Thomas Freese, The Guiding Coalition of Traditional Healers and Knowledge Keepers

**Affiliations:** 1 American Indian Health and Services, Inc., Santa Barbara, CA, USA; 2 University of California, Los Angeles, CA, USA; 3 Kauffman and Associates, Inc., Montana State University, Bozeman, WA, USA; 4 University of California, Los Angeles, Integrated Substance Use and Addiction Programs, CA, USA; 5 California Consortium for Urban Indian Health, San Francisco, CA, USA

**Keywords:** health equity, crisis response, American Indian/Alaska Native, Indigenous culture, opioid overdose, culture as health, program description, suicide, historical trauma, resilience

## Abstract

**Background:**

American Indian and Alaska Native communities are disproportionately impacted by the crises of overdose death, self-harm, and other traumas, which lead to inequities in health outcomes. Despite these inequities, American Indian and Alaska Native people have rich and varied traditional practices rooted in resiliency that promote health but have often been overlooked, excluded, or dismissed entirely in Western medicine.

**Methods:**

This paper describes *Cultural Guideposts of Health*, a crisis response evaluation framework for California’s diverse American Indian and Alaska Native communities. In an adaptation of Yamane and Helm’s 2022 Culture-as-Health paradigm, a Guiding Coalition of Traditional Healers and Knowledge Keepers convened, both in person and online, and collaborated with a team of academic researchers and cultural advisors to develop the Cultural Guideposts of Health Framework. The Guiding Coalition included cultural leaders, youth, individuals with lived experience, health care providers, and community members throughout the state and engaged in an iterative process of Indigenous Conversation to develop a culturally adaptive and responsive crisis response model for use across diverse *Tribal* and urban American Indian and Alaska Native communities.

**Results:**

Discussion topics included Indigenous definitions of health, healing, and wellness and culture as health within domains of Indigenous Spirituality, Indigenous Cultural Practices, Indigenous World View, and Place-Based Sacred sites.

**Conclusion:**

The final product was a toolkit which includes a framework for evaluating crisis response and other evidence-based interventions that is adaptable across geography, organizational structures, service delivery models, and distinct *Tribal* contexts. Future directions include pilot testing and exploration of the framework’s utility as an evaluation tool.

## Introduction

American Indian and Alaska Natives (AI/AN) are a strong and resilient people. For millennia, they have thrived in complex communities with intricate social, cultural, and political systems. These communities endured the monumental shift that occurred after European contact and survived the targeted dismantling of health and wellness practices within and between Indigenous communities. AI/AN people are also disproportionately impacted by life-threatening trauma, overdose death, self-harm, and domestic violence, necessitating further attention be spent on culturally appropriate crisis response programs for this population.

Suicide remains a particularly urgent concern, with AI/AN individuals experiencing the highest suicide rates among U.S. minority groups—~22.1/100,000 compared to the national average of 14.5/100,000.^1,2^ Youth suicide rates are even higher, with AI/AN youth aged 15–24 dying by suicide at 2.4 times the rate of their non-Native peers.^2^ In California specifically, aggregated data from 2018 to 2021 demonstrated suicide rates of 14.8/100,000 among AI/AN youth, higher than all other racial/ethnic groups.^3^ Substance use disorders (SUD) affect ~10% of AI/AN individuals—rates 1.4 times higher than the general population—with alcohol use disorder affecting over 7% of the population.^4,5^ Substance and alcohol use are a significant concern, with AI/AN having the highest rates of drug-induced or alcohol-induced deaths in California in 2019.^6^ Co-occurring mental health conditions compound these challenges, with urban AI/AN youth in California experiencing high rates of both mental health and substance use disorders.^7^ Culturally relevant crisis response approaches are essential to decrease these concerning rates and restore holistic health and wellness in ways that honor Indigenous values and worldviews.^8,9^

Disproportionate rates of suicide and trauma among AI/AN populations stem from multiple predisposing factors, including the intergenerational impacts of colonization, forced relocation, and systematic disruption of traditional healing practices.^10,11^ Geographic isolation presents additional barriers, particularly for the 22% of AI/AN people living in rural areas, where access to behavioral health services is severely limited.^12^ With limited access and geography, the onus of responsibility for crisis response falls to limited community-based resources. Cultural barriers also play a significant role, including stigma around treatment-seeking and discomfort with traditional Western treatment approaches that often fail to align with Indigenous values and healing traditions (SAMHSA^13^; see also Tanta-Quidgeon et al.^14^). Historical trauma response manifests across generations, contributing to health disparities through complex biopsychosocial pathways.^15^ Contemporary threats to culture, language, and lifeways, and lack of access to traditional foods contribute to these risks. Coupled with other socio-political factors, there emerges a need to capitalize on the inherent strengths and knowledge systems already possessed by AI/AN communities that offer promising pathways for addressing crises.^16^ The shortage of culturally relevant approaches is particularly evident in crisis response systems, where standardized protocols frequently overlook the importance of cultural context and community resources.^17,18^

Traditional health care practices, as described by the World Health Organization, include the “knowledge, skill, and practices based on the theories, beliefs, and experiences Indigenous to different cultures. . . used in the maintenance of health as well as in the prevention, diagnosis, improvement, or treatment of physical and mental illness.” Building on Yamane and Helm’s^19^ Culture as Health paradigm, which identifies four essential modalities—Indigenous Ways of Knowing, Indigenous Cultural Practices, Place-Based Sacred Sites, and Indigenous Spirituality—this work establishes a preliminary framework for evaluating the cultural relevance of crisis response systems.^19^ The approach aligns with principles of community-based participatory research, ensuring that *Tribal* communities maintain control over how their knowledge is represented and utilized.^20,21^

Research suggests that culturally grounded interventions can significantly improve outcomes for AI/AN individuals experiencing mental health crises. Programs that center Indigenous knowledge systems demonstrated promising results in addressing suicidal ideation, SUD, and trauma responses.^13^ Examples include the Qungasvik intervention among AN youth, which reduced suicide risk factors while strengthening protective cultural factors^22^; the Lakota Circles of Hope (LCH) decreased risk behaviors while enhancing cultural connection^23^; and Drum-Assisted Recovery Therapy for Native Americans (DARTNA), demonstrated improved substance use outcomes through cultural reconnection.^5,24^ These approaches center Indigenous ways of knowing, integrate traditional practices, honor connection to place, and recognize the fundamental role of spirituality in wellness and healing.^14^

California is home to the largest population of AI/AN people in the United States, with over 720,000 individuals, 109 federally recognized tribes, and 65 tribes, still seeking recognition.^25^ This population is remarkably diverse, representing both California’s unique *Tribal* communities and individuals from tribes across North America who relocated to urban centers through federal programs.^26^ Approximately 90% of California’s AI/AN population lives in urban areas, creating unique challenges for maintaining cultural connections while navigating complex service systems.^27,28^ The state’s urban Indian health organizations serve as critical hubs for culturally responsive care, while rural *Tribal* health programs and Indian Health Services address the needs of more geographically isolated communities. Historical disruptions to traditional practices have created gaps in cultural knowledge transmission that impact community wellness and crisis response capacity.^5^ Suicide prevention programs like the “Never a Bother” campaign, a statewide youth suicide prevention initiative, partners with *Tribal* organizations to develop culturally responsive messaging and outreach efforts; however, comprehensive integration of Indigenous approaches to crisis response remains limited.^29^

California’s 109 unique federally recognized Tribes each hold their own traditional health care practices, yet collectively align on the belief that traditional practices are a fundamental element of health. Given the diversity of such cultural practices, the development and application of informed frameworks require flexibility and adaptability to address such a complex issue. The importance of culture in suicide and trauma prevention extends beyond superficial adaptations of Western models. As Yamane and Helm^19^ articulate, an Indigenous “Culture as Health” paradigm recognizes that cultural practices themselves constitute comprehensive health-promoting systems. This approach represents an evolution from early cultural adaptation efforts, which often extracted isolated elements from Indigenous cultures, toward more authentic integration of Indigenous epistemologies.^30^ Evidence suggests that deeper cultural integration yields more sustainable outcomes.^5^ When applied to crisis response, culturally centered interventions address both immediate symptoms and underlying disruptions to identity, community connection, and spiritual well-being.^9^ Research consistently demonstrates that stronger cultural identification serves as a protective factor against suicide and substance use among AI/AN populations, with cultural participation associated with reduced ideation, improved coping strategies, and enhanced resilience.^18^ This protective effect operates through multiple mechanisms, including enhanced social support, restored sense of purpose, and reconnection to cultural values that explicitly promote life and community wellbeing.^17,22^

Addressing crises in California *Tribal* communities requires approaches that honor Indigenous knowledge systems while leveraging contemporary resources.^31^ Crisis response tools must be adapted to diverse *Tribal* contexts, particularly within California’s complex landscape of urban and rural AI/AN communities.^5,32^ Implementation research indicates that community adoption of crisis response systems depends heavily on cultural congruence and local leadership, factors often overlooked in conventional service delivery models.^22,33^ Recent innovations in Indigenous evaluation methodologies offer promising frameworks for assessing the effectiveness of culturally centered interventions, moving beyond Western metrics to incorporate Indigenous definitions of success and healing.^34^

This paper describes the iterative process of developing *Cultural Guideposts of Health*, a crisis response evaluation framework created by a Coalition of Indigenous community members, traditional healers, and health allies following Indigenous methodological principles.^21,35^ Through detailed documentation of this collaborative process, we provide a framework which AI/AN-serving organizations throughout California can adapt to their specific community contexts, ultimately contributing to more effective, culturally responsive crisis intervention systems that recognize culture not merely as a factor in health, but as health itself.^9,19^

## Design and methods

The development of the *Cultural Guideposts of Health* Framework represents an advancement in addressing crisis response among *Tribal*/urban Indian communities in California. Through a collaborative process with a Guiding Coalition of Traditional Healers and Knowledge Keepers (GC) across California (described below), we developed a framework that bridges Indigenous epistemologies with contemporary healthcare practices while maintaining cultural integrity. Utilizing a culturally appropriate methodology will help ensure that the development of crisis response programs will be culturally acceptable and resonate with the diverse trial population throughout California.

Central to this co-creative process was the practice of *Indigenous Conversation*—a recognized Indigenous research method that prioritizes open, relationship-based dialogue.^21,35^ Rather than conducting linear interviews or focus groups, we honored fluid conversation that enabled participants to share cultural stories, insights, and community-specific perspectives. This relational approach aligns with what Barlo et al.^21^ describe as “yarning”—a culturally safe conversational process where knowledge is shared through storytelling, reflection, and collective meaning-making. Following community-based participatory methods^20^ and relational research structures, we viewed and understood “data collection” as the *gathering, sharing, and gifting of knowledge.* Instead of “extracting” information, we convened dialogues where *Elders*, youth, and community members shared experiences of crisis and healing. By documenting the resulting wisdom as collectively held teachings, we upheld the principle that knowledge ultimately belongs to the community. Throughout this process, we maintained protocols to protect sacred knowledge, ensuring that sensitive cultural information remained within appropriate community boundaries. As one Coalition member said, “We are not just describing programs; we are carrying our ancestors’ stories and future generations’ hopes into this conversation.”

### Foundational inquiry: Defining health and healing

The GC first convened in June 2023 for in-depth discussions centered on two questions: “What is health?” and “What is healing?” Conversations revealed deeply interconnected understandings where balance, community connection, and cultural integration emerged as central themes. GC members defined health as “being balanced, looking for direction” with a “holistic perspective.” Members also emphasized the relational nature of health.

The GC also addressed difficult aspects of healing with the question, “What does the healing process look like when it’s not pretty?” Discussion revealed nuanced perspectives on inclusion, particularly regarding individuals under the influence at cultural gatherings. Rather than binary approaches of inclusion or exclusion, members described cultural protocols that maintain ceremonial integrity while honoring everyone’s healing journey.

This exploration of complex healing dynamics demonstrates how the methodology created space for culturally nuanced discussions that would not emerge in conventional research approaches. It also highlights the GC’s commitment to addressing real-world challenges in implementing culturally responsive crisis response.

### Iterative development process

**Figure 1.**
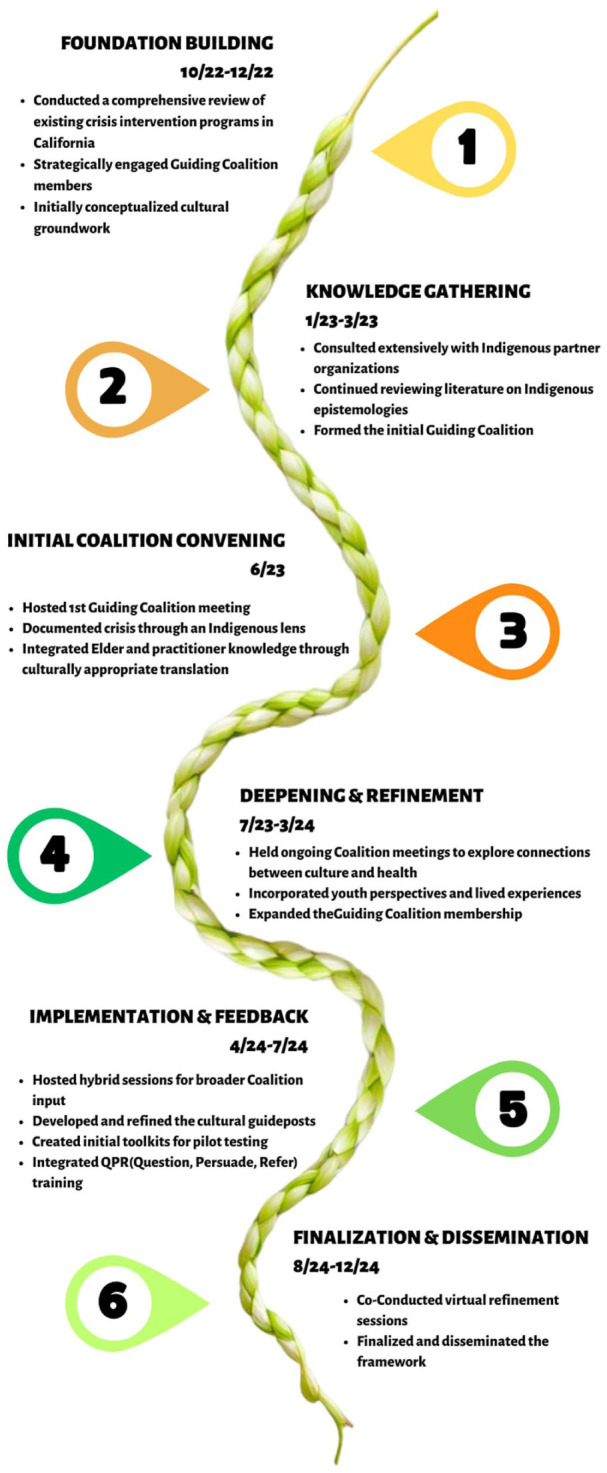
Timeline for the development of the Cultural Guideposts of Health.

Figure 1 represents the project timeline. From October 2022 to December 2024, the project proceeded through six interconnected phases: (1) Foundation Building; (2) Knowledge Gathering; (3) Initial Coalition Convening; (4) Deepening and Refinement; (5) Implementation and Feedback; and (6) Finalization and Dissemination. This iterative approach allowed for continuous refinement based on community feedback and cultural knowledge.

This approach helped clarify how each Culture as Health modality applied—or required adaptation—to meet community realities. As the GC engaged in conversation around each domain, they discussed cultural interpretations to shape crisis-response strategies and reflect both individual and communal priorities. A recurring challenge arose in attempting to compartmentalize cultural practices; Coalition members noted that culture is best understood as a unified, holistic system, rather than fragmented components.

### Theoretical framework and adaptation

The theoretical framework of our approach drew heavily on Yamane and Helm’s^19^ Culture as Health paradigm, which identifies four essential modalities. However, rather than applying this framework wholesale, the GC engaged in what one member described as a process of “deconstructing and reconstructing.” to ensure the model authentically reflected the lived experiences and cultural contexts of AI/AN communities. The Cultural Guideposts Framework emerged from a recognition that conventional crisis response models often fail to address the unique needs of AI/AN communities.^34,36^ This Coalition’s initial work focused on translating theoretical concepts into practical guidance for crisis responders working in diverse California *Tribal* communities, following principles of cultural rigor and decolonizing methodologies.^34,36^

### Critical interrogation and transformation

During the November 2023 meeting, participants critically examined the original framework categories, noting that the domain segmentation created an “unnatural” and “very linear way of looking at things.” One Coalition member observed, “Everything in traditional practice is intertwined. You can’t look at where it is separated; you have to look at it as a whole.” This insight prompted a significant reconceptualization of the framework structure.

The GC proposed fundamental modifications to better align with California Indian epistemologies. Most notably, they renamed the “Indigenous Ways of Knowing” domain to “Indigenous World View,” reflecting a more holistic understanding of knowledge and being. This shift emphasized that Indigenous perspectives encompass not just what is known, but how that knowledge is integrated into all aspects of existence. The Coalition deliberately removed terminology beginning with “de-” (such as “decolonizing”), noting that “we cannot reverse colonization, but we can work on Indigenous practices.” Instead, they replaced these terms with strength-based language like “reclaiming” that centers Indigenous agency and cultural continuity.

The group also challenged the concept of “fixed physical locale” within the Place-Based Sacred Sites domain as potentially excluding urban Indians and those disconnected from ancestral lands. They recognized that sacred spaces can be both “fixed and transitory at the same time.” As one member expressed, “Though we do not have our original location for cultural practices, we have now created one, that is, sacred to us.” This acknowledgment of contemporary Indigenous realities—especially for urban communities resulting from federal relocation policies—ensured the framework’s relevance across diverse California Indian contexts.

### Adaptability and community ownership

A central principle guiding the framework development was adaptability to diverse *Tribal* contexts. As articulated in the Cultural Guideposts of Health Reflection Guide, “The Guideposts are designed to be adaptable, encouraging you to apply these concepts in ways that resonate with your specific community and traditions. The terms provided are not definitive; they are flexible and should be molded to fit the people and places they are meant to serve.” Programs implementing these Guideposts must also commit to protecting the sacred nature of cultural knowledge shared in healing contexts.

Coalition members emphasized that the framework must accommodate both rural and urban experiences. As one member noted, “The model considers California’s landscape and the differences between urban and rural experiences. Each tribe, rancheria, or individual can use the model to evaluate evidence-based practices for addressing crises in their community, organization, or practice.”

This transformation of the Yamane and Helm model resulted in three overlapping domains—Indigenous Cultural Practices, Indigenous World View, and Place-Based Sacred Sites—with an overarching domain, Indigenous Spirituality. The resulting framework maintains the essential principles of the original model while enhancing its cultural relevance and applicability for California Indian communities.

### Implementation and feedback

#### Foundation building

Beginning in October 2022, an academic institution (lead team) engaged in a comprehensive literature review of existing crisis intervention programs in California with the intention of identifying and/or developing culturally responsive crisis intervention services in California. Traditional healers and knowledge keepers from *Tribal* and urban Indian health programs, community organizations, and those recommended by cultural consultants were engaged to participate in the project. Leaders from an urban Indian health advocacy agency, a rural Indian health board, and other *Tribal* partners made recommendations that included Chief Culture Officers at Indian health programs, *Tribal Elders*, and those known in their communities to practice traditional healing. Members of the lead team arranged calls with potential members to discuss the project and answer questions, with the intention of engaging a diverse group in terms of geography, Tribal affiliation, gender, and lived experience.

#### Knowledge gathering

January through May 2023, the initiative continued as the lead team consulted extensively with Indigenous partner agencies in California. The team continued to review literature on Indigenous epistemologies and engaged and convened the initial Guiding Coalition of Traditional Healers and Knowledge Keepers (GC) for this project.

The final group of GC members are 16 people who represent tribes and areas across all regional parts of California: Northern (2), Capital (4), Bay Area/Mid-State (7), and Southern California (3). Efforts to ensure that knowledge keepers from both rural (9) and urban (7) areas throughout California were included. All but four members are affiliated with California Tribes. Those whose affiliations are from outside California have lived and worked in California for more than two decades. All members received an honorarium for meeting preparation and attendance.

#### Coalition convening

Over 18 months, from June 2023 to December 2024, the GC gathered eight times: three hybrid meetings (in-person and online attendance), four online virtual gatherings, and one in-person training to introduce the Guideposts during a suicide prevention training. Each gathering began with a traditional opening led by a Coalition member, establishing a sacred and culturally safe knowledge-sharing space. Each meeting closed by uplifting thanks and appreciation for the ideas and contributions of each member. These practices embody the core principle that methodology itself must emerge from and reflect Indigenous ways of being and interacting. The first meeting was focused on connection, introducing the Yamane and Helm’s^19^ model and discussing the definitions of “health” and “crisis” in Native communities. As meetings continued, the members documented varied manifestations of crisis in Indigenous contexts. Discussion questions included: (1) What crisis response approaches are working in our communities? (2) How can we best serve a person in crisis? (3) How can we best serve crisis responders? (4) How can we improve conventional approaches to crisis response to better serve *Tribal* or urban Indian communities?

#### Deepening and refinement

July 2023 through March 2024, the GC continued to meet to create and refine the model. Emerging themes were woven into a coherent framework without sacrificing each community’s cultural specificity. Based on these discussions, the literature reviewed by Yamane and Helm, and consultation from Indigenous partners, we collected themes, ideas, and specifics to organize within the Culture as Health Framework as “Cultural Guideposts.” GC members also provided input on the development of a logo for the project and the structure of the Cultural Guideposts of Health Framework. The Guideposts were designed to be approachable for all community members, avoiding academic jargon that might impede local adaptation.

This phase also worked to incorporate youth perspectives and people with lived experiences as members of the GC. Existing Coalition members and other contributors sought out additional individuals to serve as Coalition members, identifying a college student, a community advocate, a talking circle facilitator, and an additional Indigenous scholar to the GC.

#### Implementation and feedback

April through July 2024, the GC convened in hybrid sessions to engage in broader Coalition input. In accordance with Indigenous values of reciprocity, near-final versions of the Guideposts were returned to communities for feedback. Coalition members distributed the draft Toolkit for pilot testing and invited community stakeholders to review, adapt, and refine the evolving framework, ensuring it resonated with each tribe’s unique experiences and avoided a one-size-fits-all approach.

During this time period, a GC member who is also a trainer delivered an in-person suicide prevention training, *Question, Persuade, Refer (QPR)*, for Indigenous-serving health care providers in California. Training attendees used the Toolkit to determine if QPR included the Guideposts of health and which essential Guideposts should be included to be most responsive to the cultural needs of their communities. The session yielded valuable insights into both the framework’s utility and the limitations of standardized crisis response approaches. Training attendees noted that while QPR offered structured intervention steps, it lacked integration with cultural practices fundamental to healing in Indigenous communities. One participant observed that QPR “works better” than other standardized approaches because “it’s not scripted,” allowing for cultural adaptation. However, they emphasized that “the biggest thing is to know your community,” underscoring the importance of place-based knowledge and community relationships.

#### Finalization and dissemination

From August through December 2024, the GC co-conducted virtual refinement sessions. Three GC members and one UCLA staff member did a conference presentation for stakeholders throughout California.

During these meetings, GC members provided substantive feedback on both content and visual representation. They emphasized the importance of visually representing the interconnectedness of the domains and utilizing circles rather than rectangles. Members also recommended enhancing the reflection questions to be more open-ended and action-oriented. They suggested including case examples demonstrating the application of the Guideposts to specific interventions, providing concrete models for implementation. This cyclical process demonstrated collective ownership and the consistent return to community for validation; refinement maintained fidelity to Indigenous methodological principles of relationality and accountability.

## Results

Through systematic analysis of Coalition dialogues and iterative refinement, we developed the Cultural Guideposts of Health Framework. Upon completion of the Framework, a subsequent Toolkit was created, which includes the Framework Trifold, a Reflection Guide, and a Program Summary. Each document, individually, provides important concepts and topics for consideration while collectively, this Toolkit offers a tangible set of guidance to apply to crisis intervention programs to better support their refinement into culturally responsive interventions for California Indian country. The Toolkit can be found in Supplemental Materials.

### Cultural Guideposts of Health Framework

**Figure 2.**
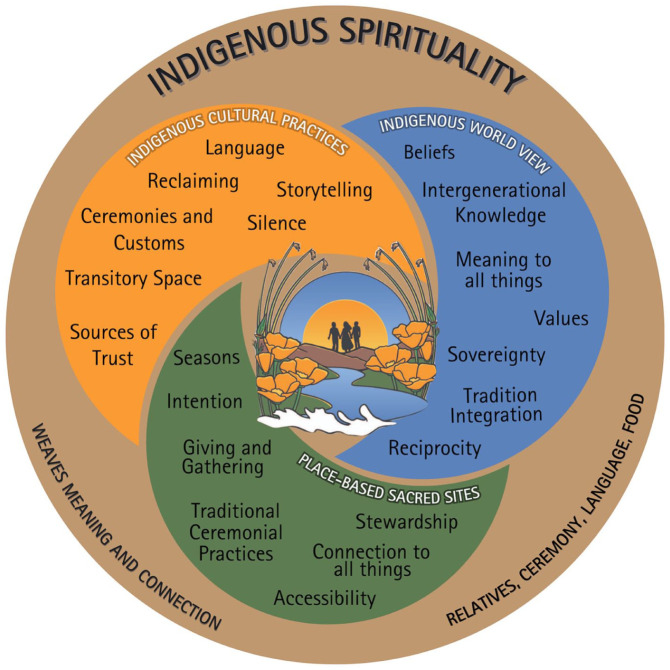
Cultural Guideposts of Health Framework.

The framework is organized into three primary, overlapping domains: Indigenous Cultural Practices, Indigenous World View, and Place-Based Sacred Sites, each containing specific Guideposts that emerged consistently across diverse *Tribal* perspectives. An overarching sphere also emerged: Indigenous Spirituality, which weaves meaning and connection throughout all elements. The Cultural Guideposts of Health Framework is shown in Figure 2.

#### Domain 1: Indigenous cultural practices

The Indigenous Cultural Practices domain encompasses elements fundamental to cultural transmission and engagement. Analysis of Coalition discussions identified seven key components within this domain: Language, Reclaiming, Storytelling Traditions, Ceremonies and Customs, Sources of Trust, Silence, and Transitory Spaces.

#### Domain 2: Indigenous world view

Within the Indigenous World View domain, Coalition members articulated seven central elements that shape Indigenous approaches to health and wellness: Values, Beliefs, Intergenerational Knowledge, Sovereignty, Meaning to All Things, Tradition Integration, and Reciprocity.

#### Domain 3: Place-based sacred sites

The Place-Based Sacred Sites domain revealed seven primary components essential to Indigenous wellness: Seasons, Intention, Traditional Ceremonial Practices, Stewardship, Connection to All Things, Gathering, and Accessibility. This domain highlighted the inseparable relationship between Indigenous wellness and connection to land and place, whether in rural or urban contexts.

#### Indigenous spirituality

Indigenous spirituality serves as the thread that weaves meaning and connection into every aspect of life, encompassing all three domains. It is not confined to a particular time or place but represents a constant presence guiding actions, thoughts, and relationships. Spirituality within this framework encompasses relatives that extend beyond the nuclear family to include the wider community and all living beings.

### Culture Guideposts of Health Toolkit

The Cultural Guideposts of Health Toolkit serves as a structure for implementation and was developed by the GC to further define and explain the individual components of each domain and provide additional context into how the concepts can be used/applied to individual communities. The Toolkit includes the following components:

1. Cultural Guideposts of Health overview
2. Cultural Guideposts of Health Reflection Guide
3. Cultural Guideposts of Health Framework trifold

Together, these materials define and provide a layout wherein interested individuals may implement culturally responsive interventions which reflect the community’s own priorities and capitalize upon their unique strengths to address crises. Implementation of these Guideposts and Health Toolkit requires ongoing commitment to protecting the sacred nature of Indigenous knowledge systems and ensuring that cultural teachings are shared only within appropriate contexts and with proper protocols.

## Discussion

The Cultural Guideposts of Health Framework and Toolkit represent a significant advancement in conceptualizing and implementing crisis response systems for AI/AN communities in California. This work emerges at a critical juncture when conventional approaches have proven insufficient to address the disproportionate rates of suicide, substance use disorders, and related crises affecting AI/AN populations and at a unique time as California begins to embark upon integration of traditional practice into the organized delivery system. This framework is particularly timely as it aligns with California’s Section 1115 Medicaid waiver to initiatives, which provide states with flexibility to evaluate novel approaches to Medicaid service delivery. Through these waivers, California has an unprecedented opportunity to incorporate culturally-specific approaches into the healthcare system, including traditional healing practices for AI/AN communities. The Framework offers a structured, evidence-informed approach that can inform waiver implementation by providing clear pathways for integrating Indigenous approaches within Medicaid-funded services. By supporting Section 1115 waiver implementation with culturally grounded frameworks, this work helps bridge the gap between policy intentions and meaningful service delivery that respects Indigenous healing traditions while meeting the requirements of healthcare systems.

### Cultural Guideposts of Health primary applications

The framework offers four primary practical applications across diverse settings in California Indian Country. First, the framework provides a structured approach for assessing existing services through cultural lenses, with specific attention to the interaction between Indigenous knowledge systems and contemporary healthcare practices. This assessment process goes beyond surface-level cultural adaptations to examine fundamental alignments and misalignments between crisis response and cultural values. Second, it offers a methodological foundation for designing culturally grounded interventions that maintain integrity with both Indigenous and clinical approaches. Third, it establishes criteria for evaluating evidence-based practices for cultural appropriateness and adaptation potential. Finally, it provides guidance for program development that centers Indigenous ways of knowing while engaging with contemporary healthcare systems.

### First: Structured assessment of existing services

The Toolkit provides a structured approach for examining and adapting services while centering Indigenous values. By posing the central questions, “Does the intervention, approach, or program include these Guideposts of health? What is present? What essential Guideposts are missing?” communities can systematically evaluate whether crisis response systems honor their cultural contexts. This assessment process transcends surface-level cultural adaptations to consider deeper structural alignment and services that accommodate extended healing processes rather than brief interventions and highlight the need for crisis response systems that accommodate Indigenous understandings of time, healing, and recovery.

### Second: Culturally grounded intervention/service design

The methodological foundation engaged was critical to developing a culturally grounded framework that honors Indigenous perspectives while meeting practical implementation needs. As articulated by a GC member, the intention is to “Integrate Western approach into the foundations of culture, not the opposite.” This structure emerged organically through a collaborative, community-driven process involving knowledge keepers, *Tribal* healthcare providers, and community members; we were able to identify Cultural Guideposts that transcend superficial adaptations and instead reflect core Indigenous values and healing approaches. This methodology ensured that the resulting framework wasn’t simply a Western model with cultural elements attached, but rather a genuinely Indigenous approach that selectively incorporates compatible Western practices. The iterative refinement process, which included multiple rounds of feedback from *Tribal* knowledge keepers across California, resulted in an adaptable program that helps meet the unique needs of diverse *Tribal* communities while maintaining fidelity to shared cultural values. By prioritizing Indigenous voices throughout the research and development process, a framework was created that resonates with lived experiences and traditional knowledge systems while remaining practical for implementation within existing healthcare structures.

### Third: Evidence-based practice evaluation

Our findings demonstrate a mechanism for incorporating a “Two-Eyed Seeing” approach incorporating Indigenous knowledge systems and the strengths of western-based knowledge.^37^ Indigenous and contemporary health practices are incorporated by maintaining cultural integrity—addressing what Yamane and Helm’s^19^ term “epistemic exploitation” and ensuring that evaluation methods honor Indigenous knowledge systems. The Framework establishes specific criteria for evaluating evidence-based practices for cultural appropriateness and adaptation potential by providing concrete yet flexible metrics for determining whether and how evidence-based practices might be adapted. This addresses a persistent challenge in implementation science, where interventions with strong empirical support often lack cultural relevance for AI/AN communities.^33^

### Fourth: Indigenous centered program development

The framework provides guidance for Indigenous-centered program development to help communities and organizations develop crisis response approaches that authentically reflect local cultural contexts while meeting institutional requirements for funding and sustainability. One GC member emphasized that “you’re not the expert, the community’s Healers are the experts.” The framework supports this principle by providing a structure through which local knowledge keepers can define priorities and practices for crisis response while maintaining appropriate safeguards for sacred knowledge.

### Framework adaptability

The framework demonstrates adaptability across multiple dimensions, including:

1. *Geographic context (rural/urban settings)*: The Guideposts acknowledge different relationships to place and community in rural, reservation, rancheria, and urban settings. As one member noted, “Rez and urban Indians are different. Urban will be into hotlines, Rez Indians want cultural things (songs, fires). There has to be a differentiation between the two.”
2. *Organizational structure (Tribal programs, urban Indian organizations, mainstream healthcare providers)*: One Coalition member described the ideal: “What would be great if we had a traditional healer with their own office, full benefits, actively involved in treatment team meetings.”
3. *Service delivery models (individual practice, program-level implementation, system-wide integration)*: The Guideposts can inform individual practitioner approaches, program-level policies, or system-wide transformations in crisis response. They address both immediate crisis intervention and longer-term healing processes.
4. *Cultural specificity (Tribal-specific protocols, pan-Indian approaches, hybrid models)*: It acknowledges that “different communities should be empowered to identify what works for them.”

This adaptability ensures that the Framework can serve as a living framework, responsive to the dynamic nature of both crisis response needs and cultural practices in California Indian Country.

### Future directions

This framework opens the following avenues for future development:

1. *Specific applications*: Further exploration of how the Guideposts interface with relevant crisis scenarios, including opioid and stimulant use interventions, harm reduction, prevention, and recovery support services.
2. *Pilot testing*: Implementation of Indigenous health practices for crisis prevention based on the framework.
3. *Evaluation tool*: Use of the Guideposts as an evaluation tool for culturally defined best practices, potentially informing policy initiatives like the 1115 waiver.
4. *Broader applications*: While focused on California AI/AN people, the methodology and framework have potential applications for other Indigenous populations and settings.

There are several potential limitations of the framework. The heterogeneity of California’s *Tribal* communities may require additional adaptation to address specific cultural contexts and traditions. While the framework was designed to be flexible, further work is needed to validate its effectiveness across different *Tribal* settings and healthcare environments. Additionally, structural barriers, including funding constraints, workforce shortages in *Tribal* communities, and policy restrictions, may challenge full implementation in some contexts. The framework also requires ongoing engagement with knowledge keepers and traditional healers whose expertise may not be readily accessible in all communities, along with protocols to protect sacred knowledge from inappropriate use or commercialization. Finally, the evaluation of outcomes requires careful consideration of Indigenous metrics of success that may differ from conventional healthcare measurements. Future research should address these limitations through collaborative approaches that continue to center Indigenous ways of knowing.

## Conclusion

Development of the Cultural Guideposts of Health Framework represents a significant advancement in how crisis response systems can be conceptualized and implemented for AI/AN communities in California and offers a transformative approach that integrates Indigenous wisdom with contemporary healthcare practices. Our findings demonstrate the feasibility and value of a collaborative, community-driven process that centers Indigenous knowledge systems while creating practical tools for implementation across diverse settings. This Toolkit ultimately provides a community-driven resource for crisis response in California Indian Country that recognizes culture not merely as a factor in health, but as health itself.^9,19,31^ By centering Indigenous knowledge systems and providing practical tools for implementation, this framework empowers communities to develop culturally responsive solutions that address the root causes of crises while honoring traditional healing practices. As *Tribal* communities continue to face disproportionate impacts from substance use and other mental health crises, this culturally grounded approach provides a critical foundation for building resilient systems of care that can effectively support recovery and wellness in ways that resonate with Indigenous values and worldviews.

Central to all applications of this framework is the imperative to protect Indigenous knowledge through appropriate cultural protocols and community oversight. Future intentions include the development of a dissemination strategy as well as an implementation plan to ensure materials are available online and in print form. Plans also include training and learning collaboratives across the state to support successful delivery to clinics and agencies, as well as application for opioid and stimulant use, including interventions, harm reduction, prevention, and recovery support services to enhance culturally appropriate service delivery.

## Supplemental Material

sj-docx-1-phj-10.1177_22799036251410263 – Supplemental material for Cultural Guideposts of Health: A crisis response evaluation framework for California’s diverse Indigenous communities Supplemental material, sj-docx-1-phj-10.1177_22799036251410263 for Cultural Guideposts of Health: A crisis response evaluation framework for California’s diverse Indigenous communities by Krista Armenta-Belen, LittleDove Rey, Joshua Severns, Daniel Dickerson, Virginia Hedrick, Gloria Miele, Mamta Bhakta, Beth Rutkowski and Thomas Freese in Journal of Public Health Research
