# Supplementary material for: Cultural Guideposts of Health: A crisis response evaluation framework for California’s diverse Indigenous communities
Source: J Public Health Res. 2026 Jan 20;15(1):22799036251410263. doi: 10.1177/22799036251410263 (PMC12820021; doi:10.1177/22799036251410263)
Supplement: sj-docx-1-phj-10.1177_22799036251410263 – Supplemental material for Cultural Guideposts of Health: A crisis response evaluation framework for California’s diverse Indigenous communities [file sj-docx-1-phj-10.1177_22799036251410263.docx]

# Supplementary Item: Indigenous Conversational Guide for Guiding Coalition Sessions

**Title:** Indigenous Conversation Guide for “Culture is Health” Guiding Coalition Discussions
**Purpose:** This guide outlines the open-ended questions and prompts used to facilitate group dialogues with the Guiding Coalition of Traditional Healers and Knowledge Keepers. Instead of a formal interview or focus group questionnaire, a culturally grounded conversational approach was used. The questions below served as starting points for discussion, allowing participants to share stories, reflections, and collective knowledge in a fluid, respectful manner. Each prompt was explored in circle conversations, with the facilitator and members encouraging storytelling and deep dialogue. (Note: The discussions were iterative; not every question was asked at every meeting, but over the course of the gatherings these topics were all addressed. The conversational style meant participants could guide the flow, and follow-up questions emerged organically from the dialogue.)

**Guiding Questions Used:**

- **“What is health?”** – (First Coalition meeting, June 2023) – This fundamental question invited each member to describe health from an Indigenous perspective, setting the stage that health encompasses balance, connection, and culture. It helped surface core values and holistic definitions of wellbeing in the community.
- **“What is healing?”** – (First meeting continuation) – A companion to the above, this question prompted discussion of what the healing process means in Indigenous contexts. Coalition members shared how they view healing as a journey toward balance, involving trust, tradition, and sometimes collective ceremony.
- **“How do we define ‘crisis’ in our community?”** – (Early meetings) – This prompt (discussed as part of defining terms) asked members to articulate what constitutes a crisis for their tribes/communities. It was used to contrast mainstream definitions with culturally informed understandings of crisis. (This helped ensure the framework addressed the correct problems as experienced by Native communities, although a direct quote is not in the text, the group did discuss the definition of “crisis”.)
- **“What crisis response approaches are currently working in our communities?”** – (Initial Coalition Convening phase) – This question focused on positives: identifying existing programs or practices (whether traditional or Western) that coalition members felt were effective in responding to mental health or substance-related crises in their communities. It encouraged sharing success stories and elements to emulate.
- **“How can we best serve a person in crisis?”** – (Coalition Convening dialogues) – This prompt guided discussion on culturally appropriate ways to help an individual who is experiencing a crisis. It elicited perspectives on immediate response tactics, traditional support systems, and the role of family/community in aiding someone in acute distress.
- **“How can we best serve the crisis responders?”** – (Coalition Convening dialogues) – Recognizing that those who respond to crises (e.g., clinic staff, first responders, traditional healers) also need support, this question gathered ideas on caring for the caregiver. Coalition members discussed training, self-care, and cultural grounding for responders to prevent burnout and improve effectiveness.
- **“How can we improve conventional crisis response approaches to better serve tribal or urban Indian communities?”** – (Coalition Convening dialogues) – This expansive question invited critique of mainstream crisis intervention models and brainstorming of modifications needed for cultural relevance. Discussions here highlighted gaps in Western approaches and generated ideas that fed directly into the framework’s domains (for instance, the need to integrate family and spirituality, or to adjust protocols to be more community-centric).
- **“What does the healing process look like when it’s not pretty?”** – (Deepening phase discussion) – This question was raised to explore challenging scenarios in healing, such as when individuals are struggling (e.g., under the influence of substances) while trying to engage in cultural healing practices. It prompted the group to discuss culturally nuanced protocols of inclusion and boundaries – for example, how ceremonies handle someone who is intoxicated: not turning them away from healing, but ensuring safety for others (e.g., having them sit outside the lodge). This conversation helped the coalition address real-life complexities and reinforced the importance of non-judgmental, flexible support in the framework.

(Follow-up prompts: The facilitator used natural follow-ups like “Can you share an example?” or “What about in an urban setting?” to deepen each topic. Additionally, as themes emerged, the Coalition might pose new questions to itself, such as “Which cultural teachings are most important for crisis work?” or “How do we ensure youth voice is included?” These were handled in the flow of conversation rather than as pre-written questions.)

**Usage:** These guiding questions were used in a talking-circle format in a conversational way. Everyone had opportunities to speak to each question, and discussions were allowed to flow freely, sometimes returning to earlier questions as new insights emerged. The process was iterative: answers to early questions (like definitions of health/healing) informed later discussions (like what effective crisis response entails). By using these open-ended prompts in an Indigenous “conversation” method, the facilitators ensured that the knowledge shared was participant-driven and culturally authentic, laying the foundation for the Cultural Guideposts of Health framework.

**Note:** The above questions are provided as a supplementary guide to illustrate the conversational interview technique applied in this study. They reflect the key topics that structured the Guiding Coalition’s dialogue while allowing for storytelling and relational interaction consistent with Indigenous research methodologies. This guide can be referenced by other researchers or community facilitators aiming to adopt a similar approach, with the understanding that flexibility and cultural protocols should guide any such Indigenous conversations.
